# Transcriptome Sequencing Reveals Distinct Stress Response of *Listeria monocytogenes* 6179 During Logarithmic and Stationary Growth Phases

**DOI:** 10.1002/mbo3.70140

**Published:** 2025-11-14

**Authors:** Jessica L. Strathman‐Runyan, Bienvenido W. Tibbs‐Cortes, Stephan Schmitz‐Esser

**Affiliations:** ^1^ Interdepartmental Microbiology Graduate Program Iowa State University Ames Iowa USA; ^2^ Department of Animal Science Iowa State University Ames Iowa USA; ^3^ Infectious Bacterial Diseases Unit Agricultural Research Service USDA Ames Iowa USA

**Keywords:** acid stress, *Listeria monocytogenes*, oxidative stress, transcriptome

## Abstract

*Listeria monocytogenes* is a food‐borne pathogen that continues to threaten food safety by persisting in food production environments (FPEs). Its tolerance to stressors introduced in FPEs is well characterized; however, the effect of the growth phase on stress response gene expression is less understood. Here, we utilize transcriptome sequencing to analyze gene expression of the persistent *L. monocytogenes* strain 6179 in response to 30‐min exposure to either lactic acid or oxidative stress when grown to either logarithmic or stationary growth phase. Analysis of this data revealed distinct gene expression responses to stress exposure between the two growth phases. Exposure to lactic acid (1%, pH 3.4) resulted in 1809 significant (*Q* < 0.05, log_2_fold changes with absolute values ≥ 1) differentially expressed (DE) genes in stationary phase cells and 175 significant DE genes in logarithmic growth phase cells. Upon oxidative stress exposure (15 mM hydrogen peroxide), 184 significant DE genes were observed in the stationary phase and 819 significant DE genes in the logarithmic phase. Interestingly, in the logarithmic growth phase, 37 of the 50 most upregulated genes were shared between responses to acid and oxidative stress; these included genes involved in cysteine transport. In contrast, stationary phase cell gene expression was more influenced by the type of stress exposure, and the majority of upregulated genes were members of the σ^B^ regulon. Collectively, these results provide further insight into the impact of growth phase on gene expression in *L. monocytogenes* in response to lactic acid and hydrogen peroxide stress exposure.

## Graphical abstract

Visualization of significantly enriched KEGG pathways under different stress conditions: (A) stationary phase cells exposed to 15 mM hydrogen peroxide, (B) stationary phase cells exposed to 1% lactic acid, (C) logarithmic phase cells exposed to 15 mM hydrogen peroxide, and (D) logarithmic phase cells exposed to 1% lactic acid.

## 1 Introduction

The prevalence and persistence of food‐borne pathogens in food production environments (FPEs) increase the risk of food product contamination and threaten consumer health. Food‐borne illnesses caused by these pathogens can lead to a range of symptoms, from mild gastrointestinal pain to life‐threatening infections requiring hospitalization. Severe infections resulting in death can also occur. One such pathogen is *Listeria monocytogenes*, the causative agent of the potentially deadly food‐borne illness listeriosis. Although the incidence of listeriosis cases is low compared with other food‐borne illnesses, the mortality rate is significantly higher (Scallan et al. 2011; Tack et al. 2020). The largest recorded listeriosis outbreak, occurring in 2017–2018 in South Africa, resulted in over 1060 confirmed cases of listeriosis and 216 deaths (Kaptchouang Tchatchouang et al. 2020). In addition to the health risk, listeriosis outbreaks carry a great economic burden with the estimated cost of illness ranging from 2.3 to 2.8 billion United States dollars annually in the United States of America (de Noordhout et al. 2014; Hoffman et al. 2015; Ivanek et al. 2004). Additionally, *L. monocytogenes* possesses resilience to high salinity, low temperatures, and acidic environments (Wiktorczyk‐Kapischke et al. 2021). This enables *L. monocytogenes* to persist in FPEs (Carpentier and Cerf 2011; Ferreira et al. 2014; Pasquali et al. 2018) and to contaminate ready‐to‐eat foods and refrigerated items, such as dairy and meat products. The severe risk to public health and the economic consequences of listeriosis outbreaks necessitate the prevention of consumer exposure to the pathogen.

To rid facilities and products of food‐borne pathogens like *L. monocytogenes*, food producers utilize many cleaning and preservation methods, including detergents, disinfectants, and food preservatives. Along with these applied antimicrobial stressors, FPEs expose pathogens to other unfavorable environmental conditions, such as high and low temperatures, excessively acidic and basic conditions, high salinity, and high pressure. However, despite these eradication attempts and antimicrobial stressors, multiple strains of *L. monocytogenes* have been found persisting for months or years in FPEs (Ferreira et al. 2014; E. Fox, Hunt, et al. 2011; E. M. Fox, Leonard, et al. 2011). The hardiness of *L. monocytogenes* is partly due to its robust tolerance to adverse environments and the survival mechanisms deployed upon exposure to stressors (Wiktorczyk‐Kapischke et al. 2021). These stress response systems are well‐studied and involve the modification of gene expression (Orsi et al. 2021). The growth phase that bacterial cells are in can significantly influence stress response, gene expression, and even cell survival. In *L. monocytogenes*, gene expression in the logarithmic growth phase is focused on amino acid synthesis, cell division, and energy metabolism (Veselovsky et al. 2022; Wen et al. 2011). *L. monocytogenes* cells in the stationary phase upregulate virulence genes and alternative sigma factors, such as the stress regulator σ^B^ (Jaishankar and Srivastava 2017; Orsi et al. 2021; Schwab et al. 2005). Furthermore, studies have shown that the stationary phase confers increased tolerance to various stressors in FPEs, including pressure, acid, and osmotic stress (Davis et al. 1996; Hayman et al. 2007; Orsi et al. 2021). Although baseline gene expression differences between growth phases in *L. monocytogenes* have been studied, how these growth phases affect gene expression during stress response remains largely unclear (Fiorini et al. 2008; Weeks et al. 2004; Wen et al. 2011). The aim of this study was to use transcriptome sequencing to analyze the gene expression of the persistent *L. monocytogenes* strain 6179 grown to either logarithmic or stationary phase and subsequently exposed to lactic acid or oxidative stress.

## 2 Materials and Methods

### 2.1 Bacteria Used

The strain used for this study was *L. monocytogenes* 6179, which belongs to multi locus sequence type sequence type (ST) 121 and harbors a conserved plasmid (pLM6179) of 61.2 kbp (Schmitz‐Esser et al. 2015; Tibbs‐Cortes et al. 2023). 6179 was isolated in 2000 from Irish cheeses and cheese production facilities, where it persisted from 2000 to 2008 (E. Fox, Hunt, et al. 2011; E. M. Fox, Leonard, et al. 2011; Stessl et al. 2014). *L. monocytogenes* strains of ST121 are among the most abundant strains isolated from food and FPEs and have been shown to be well‐adapted to persistence (Henri et al. 2016; Kaszoni‐Rückerl et al. 2020; Martín et al. 2014; Maury et al. 2016, 2019; Moura et al. 2016; Pasquali et al. 2018; Zhang et al. 2024). This persistence makes 6179 a relevant strain for studying the *L. monocytogenes* stress response, and its survival mechanisms have been well characterized using various methods, including transcriptome sequencing (Casey et al. 2014; Cortes et al. 2020; Harter et al. 2017; Makariti et al. 2015; Müller et al. 2013, 2014; Naditz et al. 2019; Rychli et al. 2016).

### 2.2 Experimental Stress Conditions

Previously, our group demonstrated that stationary phase *L. monocytogenes* 6179 exhibits major gene expression changes in response to exposure to 1% vol/vol lactic acid (pH 3.4) but less robust changes when exposed to 0.01% vol/vol (3 mM) hydrogen peroxide (Cortes et al. 2020). These stress conditions were replicated here with the following modifications: cultures were grown to either logarithmic or stationary growth phase before stress exposure, and the hydrogen peroxide concentration was increased to 15 mM to elicit a stronger response (Feld et al. 2012).

For stationary growth phase cells, 6179 was inoculated into 25 mL of tryptic soy broth (TSB) (BD Bacto) and incubated for 22 h at 20°C with 200 rpm shaking. The overnight cultures (7.5 mL) were centrifuged at 4696*g* for 10 min at 20°C. After centrifugation, the supernatants were discarded, and the pellets were resuspended in 5 mL sterile 1X phosphate‐buffered saline. The OD_600_ was then measured using a spectrophotometer (SmartSpec 3000, Bio‐Rad Laboratories), and cultures were adjusted to an OD_600_ value of 3.5 ± 0.2. Then, 0.5 mL of the adjusted culture was added to 4.5 mL of TSB containing either 1% vol/vol lactic acid (pH 3.4), 15 mM hydrogen peroxide, or no additional contents for controls. These cultures were all incubated for 30 min at 20°C with 200 rpm shaking. Immediately following the incubation, the tubes were pooled to maximize RNA yield. Cellular activity was halted by using a stop solution comprised of 1:10 acid–phenol:chloroform in ethanol (Hingston et al. 2017). The pooled cultures were added to 1.5 mL of stop solution prechilled to −20°C. Cells were then pelleted, frozen, and stored for RNA extractions and later use.

For logarithmic growth phase cells, a colony of 6179 was added to 125 μL TSB and homogenized through vortexing. This mixture (50 μL) was then added to 5 mL of TSB, and cultures were incubated for 18 h overnight at 20°C with 200 rpm shaking. Cultures were diluted 1:20 in TSB and grown at 20°C with 200 rpm shaking until reaching the logarithmic growth phase, determined by an OD_600_ ~ 0.4. Next, 1.5 mL of the logarithmic growth phase cultures was used to inoculate 6 mL of TSB containing either 1% lactic acid (pH 3.4), 15 mM hydrogen peroxide, or no additional contents for controls. The increased volume relative to what was used for the stationary phase was to ensure sufficient RNA yield from the less dense logarithmic phase cultures. Cells were then incubated for 30 min at 20°C with 200 rpm shaking. After the 30‐min incubation, the tubes were pooled and added to 750 μL of stop solution prechilled to −20°C. As done with the stationary phase cells, the stress‐exposed logarithmic growth phase cells were then pelleted, frozen, and stored. For both growth phases, all stress conditions and controls were performed in three biologically independent replicates with three technical replicates per biological replicate.

### 2.3 RNA Extraction, Library Preparation, and Sequencing

RNA extractions of the samples were performed according to the manufacturer's instructions using the Invitrogen Purelink RNA Mini Kit (Invitrogen). After thawing frozen pellets on ice, samples were resuspended in 600 μL of lysis buffer from the aforementioned kit, and β‐mercaptoethanol was added for a final concentration of 1%. Physical lysis was performed using a Bead Mill 24 Homogenizer (Fisher Scientific), and RNase activity was inhibited through the addition of 1 μL of superase RNase inhibitor (Invitrogen). The Turbo DNA‐Free kit (Thermo Scientific) was used for the removal of residual DNA; successful DNA digestion was verified through a PCR targeting the *prfA* gene using the primers Lip1 (5′‐GAT ACA GAA ACA TCG GTT GGC‐3′) and Lip2 (5′‐GTG TAA TCT TGA TGC CAT CAG G‐3′) (Cortes et al. 2020; Rossmanith et al. 2006). PCR cycle conditions were conducted as described in Cortes et al. (2020). The Agilent RNA Nano 6000 Bioanalyzer Assay was used to validate RNA quality, resulting in RNA integrity numbers values ≥ 8.2 for all samples. Next, the Iowa State University DNA Facility (Ames, Iowa) performed ribosomal RNA depletion using the Ribo‐Zero Kit (Illumina), library preparation using the NEBNext Ultra II Directional RNA Library Prep Kit (New England BioLabs), and sequencing using the Illumina NovaSeq. 6000 on an SP flowcell with 100 bp single‐read sequencing.

### 2.4 Sequence Analysis

First, quality checks on raw reads were performed using FASTQC v0.11.7 (Andrews 2010). BBDuk was then used for trimming bases < Q30 and Illumina adapter sequences and for filtering reads less than 75 bp (Bushnell 2022). BowTie2 within the RNASeq by expectation maximization (RSEM) software (Li and Dewey 2011) was used to map reads to the *L. monocytogenes* 6179 chromosome and pLM6179 plasmid (GenBank accession numbers CP098509.1 and CP098510.1, respectively, Tibbs‐Cortes et al. 2023). Subsequent transcript quantification was performed using RSEM with the read start position distribution option enabled. RSEM outputs of estimated counts per gene were imported into R (v4.3.3) using the tximport package (R Core Team 2024; Soneson et al. 2016). Genes with an effective length of zero were given an effective length of one to prevent errors in downstream processing (Love 2021). For differential gene expression analysis, DESeq. 2 was utilized with a multifactorial design to account for extraction batch effects. DESeq. 2 uses the Benjamini and Hochberg method to account for multiple‐testing correction and calculate *Q* values (Benjamini and Hochberg 1995). Genes with both a *Q* < 0.05 and log_2_fold changes with absolute values ≥ 1 were considered significantly differentially expressed (DE).

Gene set enrichment analysis (GSEA) was performed to determine enriched Kyoto Encyclopedia of Genes and Genomes (KEGG) pathways in response to lactic acid and hydrogen peroxide stress at both growth phases. Briefly, the KEGG pathways associated with each 6179 protein were identified by performing a Diamond search and annotation (constrained to Firmicutes) via EggNOG‐mapper v2.1.13 (Cantalapiedra et al. 2021). Next, for each DESeq. 2 comparison, significantly DE genes were ranked by descending log_2_fold change values. The KEGG pathway information and ranked DE genes were subsequently analyzed in R 4.5.1 via the package ClusterProfiler v4.16.0 (T. Wu et al. 2021). The ClusterProfiler GSEA function was employed to identify enriched KEGG pathways, utilizing 10,000 permutations and Benjamini–Hochberg multiple‐testing correction.

## 3 Results and Discussion

**Figure 1:**
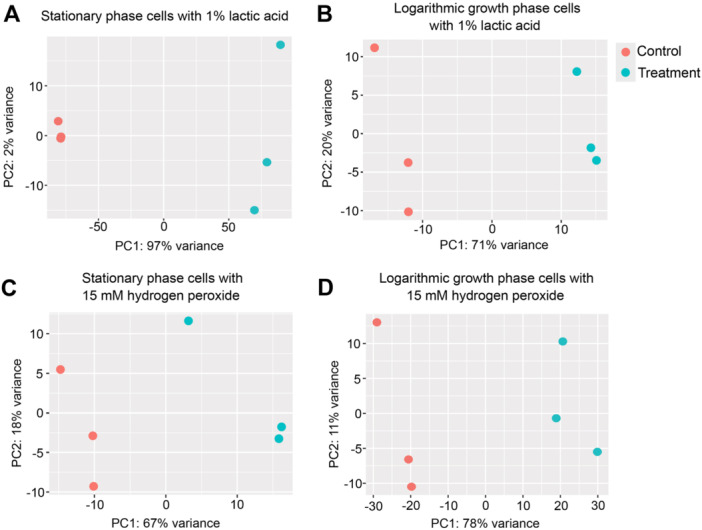
Principal component analysis plots showing differences in gene expression between replicates of *Listeria monocytogenes* 6179 in stationary and logarithmic growth phase in response to hydrogen peroxide and lactic acid stress exposure. Each point represents a single biological replicate of either control or treatment samples, with three control replicates and three treatment replicates per condition. (A) Stationary phase controls compared with stationary phase cells exposed to 1% lactic acid, (B) logarithmic growth phase controls compared with logarithmic phase cells exposed to 1% lactic acid, (C) stationary phase controls compared with stationary phase cells exposed to 15 mM hydrogen peroxide, and (D) logarithmic growth phase controls compared with logarithmic growth phase cells exposed to 15 mM hydrogen peroxide.

**Figure A1:**
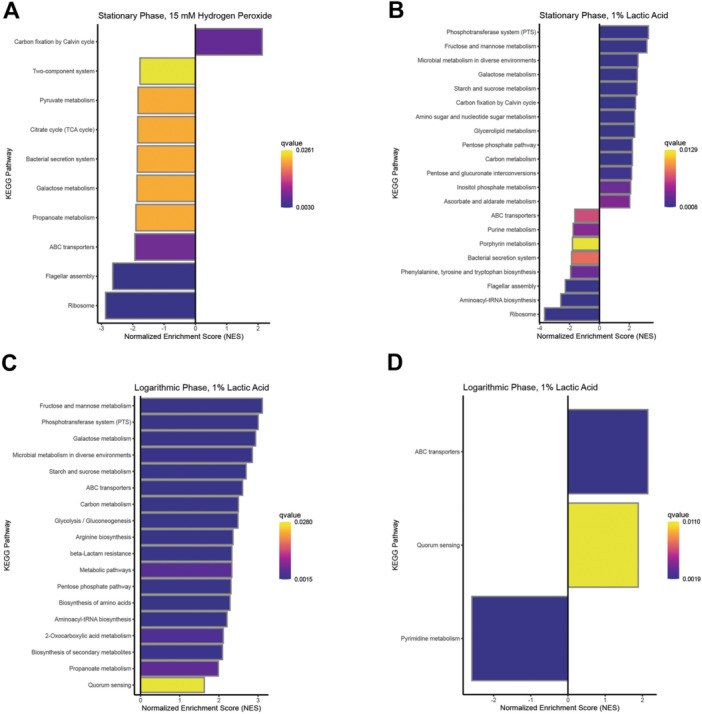
A bar chart visualization of each significantly enriched KEGG pathway per condition analyzed: (A) stationary phase cells exposed to 15 mM hydrogen peroxide, (B) stationary phase cells exposed to 1% lactic acid, (C) logarithmic phase cells exposed to 15 mM hydrogen peroxide, and (D) logarithmic phase cells exposed to 1% lactic acid. Pathways are colored by their *Q* value (Benjamini–Hochberg). The normalized enrichment score for each pathway is displayed on the *x*‐axis. Positive normalized enrichment scores indicate increased enrichment of the given pathway in the treatment versus the control. Negative scores correspond to pathways that are less enriched in the treatment compared with the control. KEGG, Kyoto Encyclopedia of Genes and Genomes.

Transcriptome sequencing resulted in 20 million to 96 million average reads per condition and an average alignment rate of 82.59% (Table 1). Principal component analysis revealed clear differences between the control and stress‐exposed replicates (Figure 1). The clustering of replicates in these plots confirms that no major outliers were present. Analysis of the transcriptome sequencing data revealed distinct and often opposing gene expression changes between the two growth phases. For stationary phase cells, 184 significant chromosomal DE genes were identified for oxidative stress, and 1809 were found for acid stress exposure (Table 2 and Supporting Information Table A1). For logarithmic growth phase cells, 819 significant DE genes were found for oxidative stress and another 175 for acid stress (Table 2 and Supporting Information Table A2). GSEA revealed that a total of 10 and 21 gene sets were enriched in stationary phase cells exposed to hydrogen peroxide and lactic acid, respectively. For logarithmic phase cells, hydrogen peroxide exposure resulted in enrichment of 18 gene sets, whereas lactic acid exposure only induced the enrichment of three gene sets (Figure A1 and Supporting Information Table A5).

**Table 1:** Average transcriptome sequencing statistics of biological triplicates for all conditions.

Growth phase, experimental condition	Average number of reads	Percent alignment rate to *Listeria monocytogenes* 6179 chromosome and plasmid (%)
Stationary phase, 1% lactic acid	28,531,553	67.39
Logarithmic growth phase, 1% lactic acid	20,091,682	87.96
Stationary phase, 15 mM hydrogen peroxide	29,140,753	79.63
Logarithmic growth phase, 15 mM hydrogen peroxide	20,131,491	88.59
Stationary phase, control	28,346,945	83.84
Logarithmic growth phase, control	96,825,226	88.10

**Table 2:** Number of significantly (*Q* < 0.05, log_2_fold changes with absolute values ≥ 1) differentially expressed (DE) chromosomal genes following 1% lactic acid and 15 mM hydrogen peroxide exposure.

Growth phase, experimental condition	Number of chromosomal DE genes	Number of upregulated chromosomal DE genes	Number of downregulated chromosomal DE genes	Log_2_fold change range
Stationary phase, 1% lactic acid	1809	873	936	−10.43 to 8.63
Logarithmic growth phase, 1% lactic acid	175	105	70	−4.16 to 6.92
Stationary phase, 15 mM hydrogen peroxide	184	83	101	−3.47 to 3.31
Logarithmic growth phase, 15 mM hydrogen peroxide	819	304	515	−8.23 to 6.75

### 3.1 Chromosomal Gene Expression Changes in Response to 1% Lactic Acid Exposure

#### 3.1.1 Stationary Growth Phase Gene Expression Patterns

**Figure 2:**
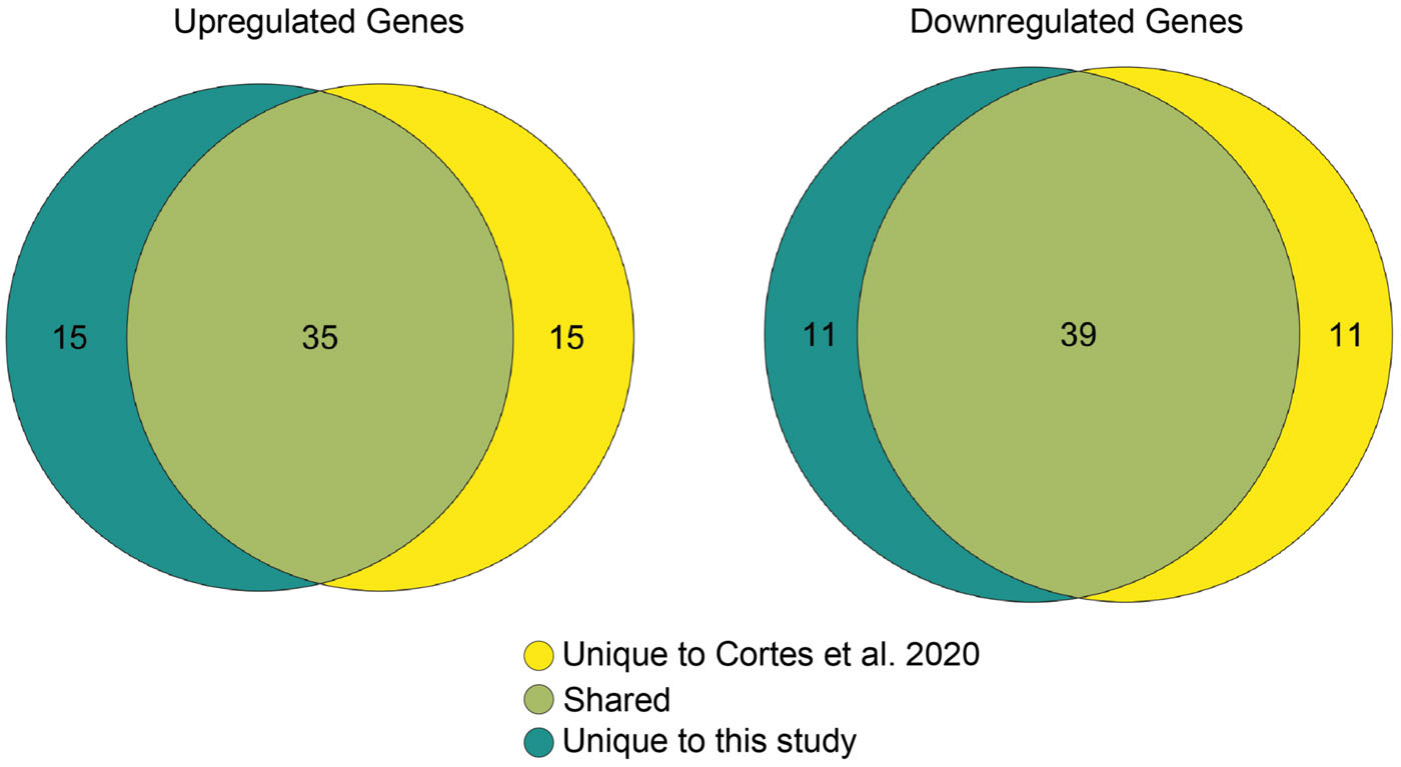
Venn diagram showing the number of unique and shared DE genes among the 50 most upregulated (left) and downregulated (right) genes in stationary growth phase cells exposed to 1% lactic acid, identified in this study and by Cortes et al. (2020).

Exposure of stationary phase 6179 cells to 1% lactic acid elicited a marked response in gene expression changes, resulting in 1809 significant DE genes with log_2_fold changes ranging from −10.43 to 8.63 (Table 2). Of these, 873 genes were upregulated, and 936 were downregulated (Table 2). The number and magnitude of log_2_fold changes of these DE genes align with what was observed in the previous study by Cortes et al. (2020), whose methods for acid stress exposure we reproduced here. Furthermore, 74% of the 50 most upregulated and downregulated genes under lactic acid exposure were shared between the two studies (Figure 2). *Lmo2213* (*LM6179_02993*), encoding a heme degradation enzyme (Duong et al. 2014), was the most upregulated gene with a log_2_fold change of 8.63, followed by *lmo0628* (*LM6179_00935*), encoding a conserved protein of unknown function, with a log_2_fold change of 8.49. The upregulation of both genes has been previously observed following lactic acid exposure (Cortes et al. 2020). Additionally, *lmo2213* has been upregulated during intracellular growth (Chatterjee et al. 2006), and *lmo0628* has been upregulated in response to pulsed light treatment (Uesugi et al. 2016). The noncoding RNA, *rli47*, was also strongly upregulated with a log_2_fold change of 7.33. Further, *rli47* was the gene with the second‐highest average normalized read counts value. This observation agrees with previous studies demonstrating high levels of *rli47* expression in response to stress exposure (Anast and Schmitz‐Esser 2020; Cortes et al. 2020; Duru et al. 2021). Transcripts of the *glmS* gene were also found in high abundance, having the ninth highest number of normalized read counts. *GlmS* encodes an enzyme used in cell wall biosynthesis (Milewski 2002), and was upregulated with a log_2_fold change of 2.52. Previous studies have found that *glmS* is upregulated following exposure to stressors, such as benzethonium chloride (Casey et al. 2014), lactic acid (Cortes et al. 2020), and high hydrostatic pressure processing (Bowman et al. 2012), suggesting a potential role in the general stress response. Additionally, virulence genes were found to be among the most upregulated genes. These included *bsh* (log_2_fold change of 6.93), which encodes a bile salt hydrolase that is important for survival of *L. monocytogenes* in the gut (Dussurget et al. 2002; Quereda et al. 2021), and internalins *inlA* and *inlB* (log_2_fold changes of 4.85 and 3.81), which are crucial for internalization into host cells (Ireton et al. 2021). *InlD* and *inlH*, additional members of the internalin multigene family with roles in pathogenicity not directly involving internalization (Personnic et al. 2010), were upregulated with log_2_fold changes of 5.63 and 7.47.

The most common and best‐characterized systems *L. monocytogenes* employs to survive acidic environments are the glutamate decarboxylase (GAD) and arginine deiminase (ADI) systems (Cotter et al. 2005; Guerreiro, Boyd, et al. 2022; Wiktorczyk‐Kapischke et al. 2021). *GadD1* and *gadT1* (*lmo447* and *lmo448*) are encoded on stress survival islet 1 (SSI‐1) (Ryan et al. 2010). However, similar to most ST121 strains, 6179 contains SSI‐2 rather than SSI‐1. SSI‐2 is an insert of two homologs of the *Listeria innocua* genes *lin0464* and *lin0465* (LM6179_00748 and LM6179_00749), which are involved in the alkaline and oxidative stress responses (Harter et al. 2017). Because of this, 6179 lacks homologs to *gadD1* and *gadT1* and only possesses homologs to *gadD2* and *gadT2*. The remaining 6179 GAD system genes (*lmo2362*, *lmo2363*, and *lmo2434*) were highly upregulated in stationary phase cells with log_2_fold changes ranging from 5.40 to 6.16. The ADI system is composed of five genes: *arcABCD* (*lmo0043* [*LM6179_00322*]), *lmo0036* (*LM6179_00315*), *lmo0039* (*LM6179_00318*), *lmo0037* (*LM6179_00316*), and *argR* (*lmo1367*; *LM6179_02110*) (Ryan et al. 2009). All five of these genes were significantly upregulated in the stationary phase with log_2_fold changes ranging from 2.59 to 4.56 for *arcABCD* and 1.02 for *argR*.

The alternative sigma factor σ^B^ is responsible for the regulation of several genes involved in stress response, virulence, and metabolism of *L. monocytognes* (Liu et al. 2019). Here, an upregulation of σ^B^‐regulated genes in stationary phase cells was also observed. *Lmo2230* (*LM6179_03010*), a gene under the regulation of σ^B^ that is often used as an indicator of σ^B^ activity (Oliveira et al. 2022; Utratna et al. 2012, 2014; J. Wu et al. 2022), was upregulated in the stationary phase with a log_2_fold change of 6.35. *Lmo2230* shares 28% amino acid identity with the arsenate reductase ArsC from *Bacillus subtilis*. However, while it is annotated as a putative arsenate reductase due to this similarity, Lmo2230 lacks the conserved essential cysteine amino acid residues that confer the functional capacity of arsenate reduction (Bennett et al. 2001; Cortes et al. 2020). While there is a lack of functional characterization of *lmo2230* that would explain its role in stress response, the upregulation of *lmo2230* in response to stress conditions is well‐evidenced. Multiple studies have shown that various stressors induce the expression of *lmo2230*, including acid stress (Cortes et al. 2020; Guerreiro, Boyd, et al. 2022; Horlbog et al. 2019), alkaline stress (Giotis et al. 2008; Wiktorczyk‐Kapischke et al. 2023), osmotic stress (Raengpradub et al. 2008; Utratna et al. 2011), cold temperatures (Hingston et al. 2017), exposure to chlorine dioxide (Pleitner et al. 2014), high‐pressure processing (Duru et al. 2021), and during desiccation of *L. monocytogenes* on steel surfaces (Kragh and Truelstrup Hansen 2020). Other upregulated genes of the σ^B^ regulon included *lmo2269* (*LM6179_03049*) and *lmo2391* (*LM6179_01880*), which were upregulated in response to lactic acid exposure with log_2_fold changes of 7.46 and 5.37, respectively. Lmo2269 and Lmo2391 are annotated as conserved hypothetical proteins, and in previous studies, both genes were upregulated during intracellular growth and under acidic stress conditions (Abram et al. 2008; Bowman et al. 2012; Chatterjee et al. 2006; Cortes et al. 2020). *Lmo2269* is also upregulated upon exposure to heat shock and ultraviolet stress (Uesugi et al. 2016; van der Veen et al. 2007).

Universal stress protein (USP) genes under σ^B^ regulation were also significantly upregulated in stationary phase cells. One such gene is the USP family gene *lmo2673* (*LM6179_00086*), which was upregulated in the stationary phase with a log_2_fold change of 6.78. Upregulation of *lmo2673* has been observed following exposure to acid, alkaline, and oxidative stressors (Bowman et al. 2012; Cortes et al. 2020; Giotis et al. 2010; Pleitner et al. 2014). In addition, the gene plays a crucial role in intracellular survival in macrophages (Chatterjee et al. 2006; Seifart Gomes et al. 2011) and is involved in damaged DNA repair (Hain et al. 2008; Wemekamp‐Kamphuis et al. 2004). *Lmo2748* (*LM6179_00162*), a USP family gene encoding general stress protein 26 (GSP26), was upregulated with a log_2_fold change of 5.93 and has been implicated in the survival of acid exposure, desiccation, and osmotic stress (Abram et al. 2008; Cortes et al. 2020; Kragh and Truelstrup Hansen 2020). Like *lmo2673*, *lmo2748* was also upregulated during the intracellular survival of *L. monocytogenes* (Chatterjee et al. 2006).

The genes encoding the manganese ATP‐Binding Cassette (ABC) transporter *mntABC* (*lmo1847* [*LM6179_02617*], *lmo1849* [*LM6179_02619*], and *lmo1848* [*LM6179_0218*]) as well as the manganese transporter *mntH* (*lmo1424*; *LM6179_2168*) were significantly upregulated with log_2_fold changes of 7.43–8.10. It has been suggested that the uptake of manganese is important to the survival of *L. monocytogenes* as manganese is an essential cofactor for many enzymes involved in stress response and carbon metabolism (Bosma et al. 2021; van Gijtenbeek et al. 2021). Indeed, the deletion of manganese transporter genes *lmo1847* and *lmo1849* resulted in a lessened stress response in *L. monocytogenes* F2365 (Liu et al. 2022). Additionally, previous studies have observed the upregulation of *mntABC* and *mntH* in *L. monocytogenes* exposed to acid stress, further supporting a functional role for these genes in the *L. monocytogenes* acid stress response (Cortes et al. 2020; J. Wu et al. 2023a).

Ribosomal proteins comprised the majority of the 50 genes with the most negative log_2_fold changes, in agreement with the negative normalized enrichment score observed for the ribosome KEGG pathway. Other downregulated genes include genes involved with flagellar biosynthesis, cell division, and transfer RNAs (tRNAs). This aligns with previous studies that also observed a downregulation of genes involved in metabolic activity and motility after acid exposure (Cortes et al. 2020; Horlbog et al. 2019).

#### 3.1.2 Logarithmic Growth Phase Response to 1% Lactic Acid

In comparison to the response of stationary phase cells to 1% lactic acid, the response of logarithmic growth phase cells to 1% lactic acid was markedly distinct. Logarithmic growth phase cells yielded a less pronounced response to lactic acid stress with 175 significant DE genes (Table 2). This was comprised of 105 upregulated genes and 70 downregulated genes with log_2_fold changes ranging from −4.16 to 6.92. Of the 20 most upregulated genes, 10 encode an operon containing an ABC transporter and its transcriptional regulator (*lmo2343*–*lmo2352*; *LM6179_03060–LM6179_03068*) with log_2_fold changes ranging from 3.64 to 6.22. In addition to the differential gene expression, GSEA demonstrated enrichment of KEGG pathways related to ABC transporters, including these genes (Figure A1 and Supporting Information Table A5). Lmo2343–Lmo2352 share 52%–75% amino acid identity with the products encoded by the *B. subtilis ytmI* operon, which is under control of the transcriptional regulator, YtlI (Burguière et al. 2005). The ABC transporter TcyKLMN (*lmo2346–lmo2349*) encoded within the *ytmI* operon functions as a cysteine/cystine importer and contributes to the activation of virulence genes in *L. monocytogenes* (Brenner et al. 2022; Burguière et al. 2005). The remaining genes encode monooxygenases (*lmo2343* and *lmo2345*), a glutaredoxin (*lmo2342*), an acetyltransferase (*lmo2350*), and an flavin mononucleotide reductase (*lmo2351*) (Brenner et al. 2022; Burguière et al. 2005). The other 10 of the 20 most upregulated genes had log_2_fold changes ranging from 3.64 to 6.92 and include genes encoding multiple ABC transporters and an OsmC family protein (encoded by *lmo0903*; *LM6179_01218*), a peroxiredoxin commonly induced by organic hydroperoxides (Ruhland and Reniere 2019). The *osmC* gene is typically induced by the presence of oxidative stress, but its upregulation has also been observed in response to osmotic stress (Ribeiro et al. 2014), high‐pressure processing (Jofré et al. 2007), and during intracellular growth (Chatterjee et al. 2006). However, the exact function of OsmC family proteins in response to these other stressors, including acid stress, is not well understood. *Lmo0135* (*ctaP, LM6179_00429*), *lmo0136* (*ctpP1*, *LM6179_00430*), and *lmo0137* (*ctpP2*, *LM6179_00431*) were upregulated with log_2_fold changes of 6.20, 5.55, and 4.72, respectively. These three genes encode transporters also involved with cystine import and are important for the pathogenicity, acid stress response, and membrane permeability of *L. monocytogenes* (Vaval Taylor et al. 2023; Xayarath et al. 2009). The remainder of the 50 most upregulated genes had log_2_fold change values of 2.63 or less.

In the transcriptome of logarithmic growth phase 6179 exposed to lactic acid, *gadT2* (*lmo2362*; *LM6179_03081*) was upregulated with a log_2_fold change of 1.52. Notably, *gadT2* was the only significantly upregulated member of the 6179 GAD system. Plainly, logarithmic growth phase data did not follow the typical gene expression associated with acid exposure of stationary phase cells. This is further demonstrated by the downregulation of *lmo0596* (*LM6179_00901*), which had a log_2_fold change of −2.02 and was one of the most downregulated genes in the logarithmic growth phase data set. This is noteworthy as *lmo0596* is under σ^B^‐regulation, and previous studies have observed its upregulation in low pH environments in both stationary and logarithmic growth phase cells (Cortes et al. 2020; Guerreiro, Boyd, et al. 2022; Guerreiro, Pucciarelli, et al. 2022). In a previous study, peak σ^B^ activity was recorded during the stationary phase (Utratna et al. 2014). With both the 6179 GAD system and *lmo0596* being σ^B^‐regulated, this may explain their lessened expression during the logarithmic growth phase. Additionally, in other *L. monocytogenes* strains, GAD activity is notably increased during the stationary phase (Cotter et al. 2001). These changes in σ^B^ and GAD activity throughout growth could have contributed to the different gene expression patterns exhibited by logarithmic growth phase cells in comparison to stationary phase cells.

#### 3.1.3 Comparison of Logarithmic and Stationary Growth Phase Gene Expression

**Figure 3:**
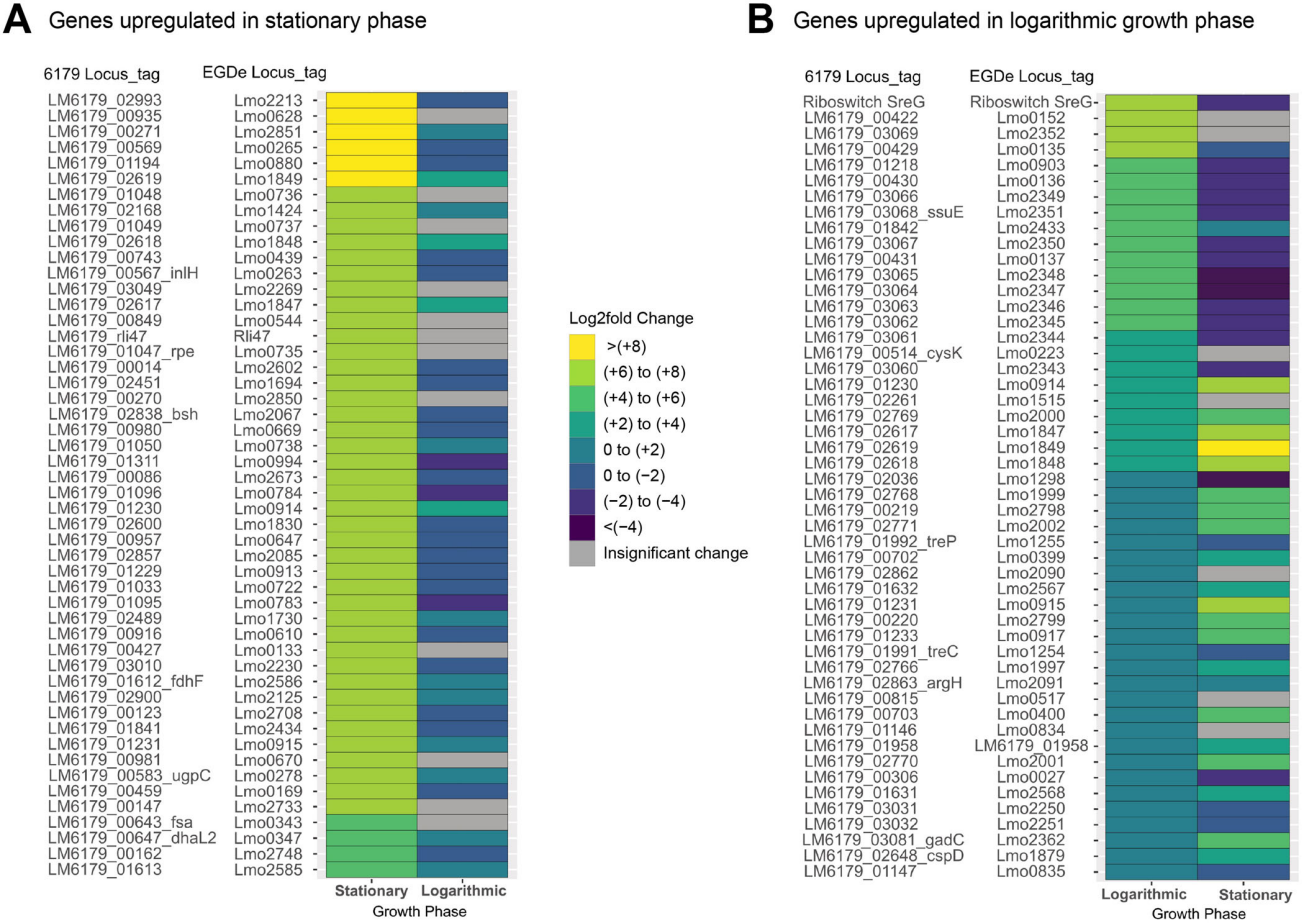
Heatmaps comparing the log_2_fold changes of significant (*Q* < 0.05 and log_2_fold changes with absolute values ≥ 1) DE genes in response to 1% lactic acid stress exposure between logarithmic and stationary growth phases. Genes are designated with *Listeria monocytogenes* EGD‐e locus tags and *L. monocytogenes* 6179 locus tags when applicable. Log_2_fold changes of these genes are displayed for both logarithmic and stationary phase cells in response to lactic acid. Genes shown in gray did not exhibit significant changes in expression. (A) Log2fold changes of the 50 most upregulated genes in stationary phase cells upon lactic acid exposure and their corresponding log_2_fold changes in logarithmic growth phase cells. (B) Log_2_fold changes of the 50 most upregulated genes in logarithmic growth phase cells upon lactic acid exposure and their corresponding log_2_fold changes in the stationary phase. DE, differentially expressed; EGD‐e, *L. monocytogenes* strain EGD‐e.

In addition to the difference in the number of DE genes between the stationary and logarithmic growth phases, there were also stark differences in which genes were significantly DE and, in some cases, in the directionality of shifts in expression. This is exemplified when comparing the log_2_fold changes of the 50 most upregulated genes in the stationary phase in response to 1% lactic acid exposure to the log_2_fold changes of the same genes in the logarithmic growth phase under the same conditions (Figure 3A). A similar pattern is also observed in the direct comparison of the log_2_fold changes of the 50 most upregulated genes in logarithmic growth phase cells to the log_2_fold changes of the same genes in stationary phase (Figure 3B). Of all significantly DE genes in response to acid exposure, 152 of these genes were upregulated in one growth phase but downregulated in the other. For example, the USP‐encoding gene *lmo2673* was downregulated in the logarithmic growth phase with a log_2_fold change of −1.07 but upregulated in the stationary phase with a log_2_fold change of 6.93. As previously described, *lmo2673* is upregulated in response to high and low pH stress (Bowman et al. 2012; Cortes et al. 2020; Giotis et al. 2010) and is critical for intracellular survival of *L. monocytogenes* (Chatterjee et al. 2006; Seifart Gomes et al. 2011).

The *ytmI* operon, including the genes encoding the cysteine/cystine transporter TcyKLMN, was also inversely expressed. In the logarithmic growth phase, these genes were upregulated with log2fold changes ranging from 3.64 to 6.22, but they were downregulated in the stationary phase cells with log2fold changes ranging from −4.88 to −2.93. Similarly, *lmo0135*, *lmo0136*, and *lmo0137*, which encode the aforementioned cystine transporters, were upregulated (log_2_fold changes of 4.72–6.20) in logarithmic growth phase but were downregulated (log_2_fold changes of −2.36 to −1.84) in stationary phase. The acquisition of cysteine via these proteins is critical for the synthesis of glutathione, an important activator of the key virulence regulator PrfA (Brenner et al. 2022). Because unregulated PrfA activity has been shown to reduce the growth rate and fitness of *L. monocytogenes* cells in both broth culture and under stress conditions (Bruno and Freitag 2010; Gaballa et al. 2019; Vasanthakrishnan et al. 2015), PrfA activity must be tightly regulated. This can be accomplished through modulation of glutathione levels, and existing evidence suggests that σ^B^ directly and/or indirectly contributes to this process (Gaballa et al. 2019). Indeed, *prfA* expression is in at least some cases negatively correlated with σ^B^ activity (Gaballa et al. 2019; Ollinger et al. 2009; Orsi et al. 2021; Vasanthakrishnan et al. 2015), the latter of which increases during logarithmic phase and reaches maximal expression during stationary phase (Utratna et al. 2014). Therefore, the observed pattern of cysteine/cystine transport gene expression here likely reflects reduction of PrfA activity through the downregulation of the genes important to glutathione availability. This may also represent a σ^B^‐independent manner of glutathione modulation, as none of these genes have previously been identified as members of the σ^B^ regulon (Liu et al. 2019).

Further demonstrating the differing roles of σ^B^ between the two growth phases, the number of σ^B^‐regulated genes also differed greatly between the logarithmic and stationary phases. Of the 50 most upregulated genes in the stationary phase, 29 genes were part of the σ^B^ regulon, while only three of the 50 most upregulated genes in the logarithmic growth phase were under σ^B^ regulation. Although σ^B^ contributes to acid stress survival during both growth phases, σ^B^ activity peaks during the stationary phase (Orsi et al. 2021; Raengpradub et al. 2008; Utratna et al. 2014) and is required for ideal cell fitness during the transition into the long‐term stationary growth phase (Bruno and Freitag 2011). During the stationary phase, σ^B^ regulates numerous genes involved with different metabolic pathways, virulence, and stress response (Orsi et al. 2021). As expected based on the literature, we observed that σ^B^ regulon genes such as those encoding the key acid resistance GAD and ADI systems were upregulated during the stationary phase and downregulated during the logarithmic phase following lactic acid exposure. σ^B^‐independent genes such as *mntABC*, however, typically had the same directionality of expression regardless of growth phase. Considering the 50 most upregulated genes during lactic acid exposure for both logarithmic and stationary growth phases, 28 σ^B^‐independent genes shared directionality of expression compared with only three σ^B^‐dependent genes. These observations combined with what is understood about the role of σ^B^ in *L. monocytogenes* suggest that the changes in σ^B^ activity throughout growth are a driving factor behind many of the differences in gene expression observed between logarithmic and stationary growth phases.

### 3.2 Gene Expression Changes in Response to 15 mM Hydrogen Peroxide

#### 3.2.1 Stationary Phase Gene Expression Patterns

**Figure 4:**
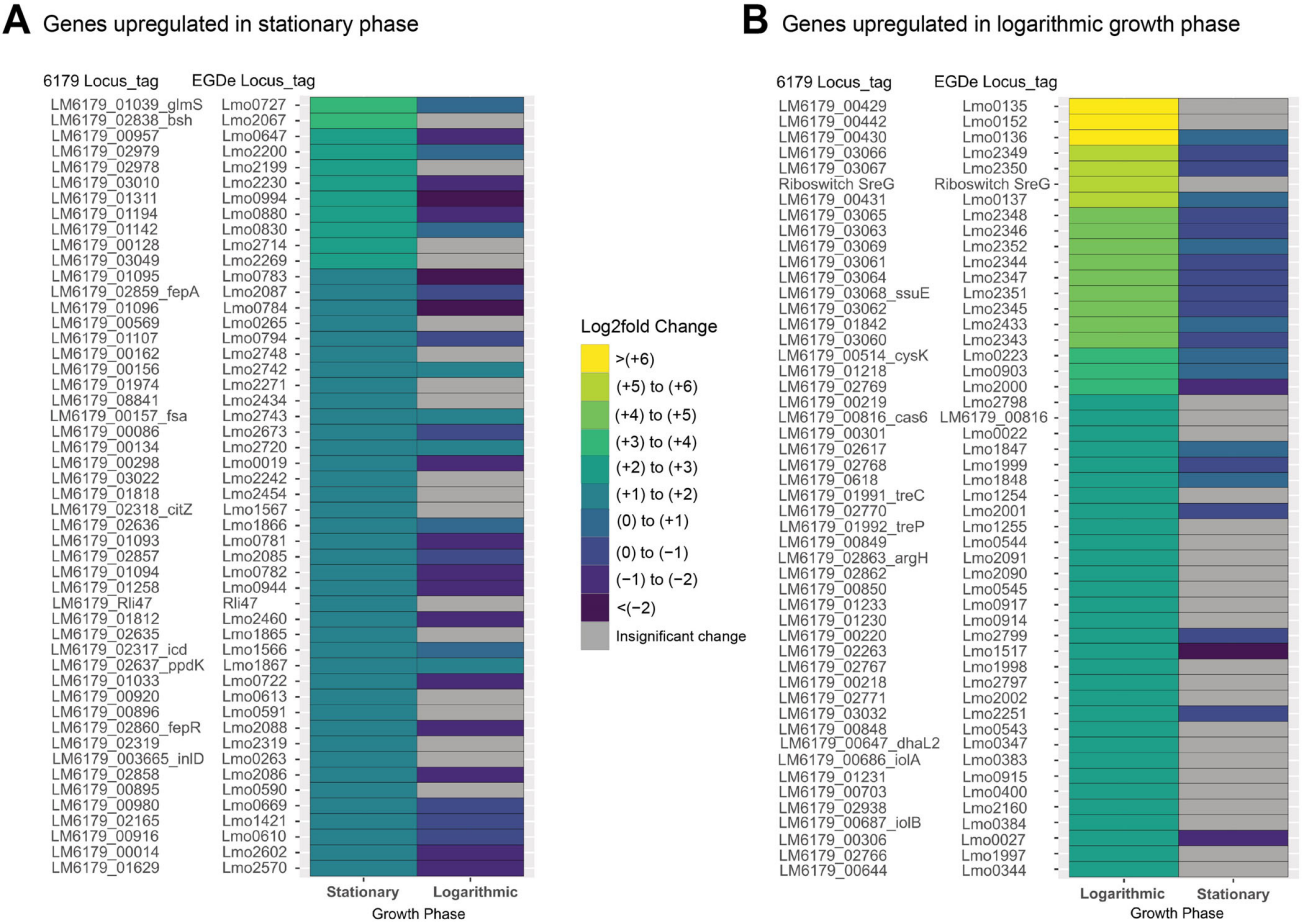
Heatmaps comparing the log_2_fold changes of significant (*Q* < 0.05 and log_2_fold changes with absolute values ≥ 1) DE genes in response to 15 mM hydrogen peroxide stress exposure between logarithmic and stationary growth phases. Genes are designated with *Listeria monocytogenes* EGD‐e locus tags and *L. monocytogenes* 6179 locus tags when applicable. Log_2_fold changes of these genes are displayed for both logarithmic and stationary phase cells in response to hydrogen peroxide. Genes shown in gray did not exhibit significant changes in expression. (A) Log_2_fold changes of the 50 most upregulated genes in stationary phase cells upon hydrogen peroxide exposure and their corresponding log_2_fold changes in logarithmic growth phase. (B) Log_2_fold changes of the 50 most upregulated genes in logarithmic growth phase cells upon hydrogen peroxide exposure and their corresponding log_2_fold changes in the stationary phase. DE, differentially expressed; EGD‐e, *L. monocytogenes* strain EGD‐e.

Oxidative stress from exposure to 15 mM hydrogen peroxide resulted in a less dramatic gene expression response in stationary phase cells than did lactic acid exposure. 184 significant DE genes were identified. Of these, 83 were upregulated and 101 were downregulated with log_2_fold change values ranging from −3.47 to 3.31 (Table 2 and Figure 4). As expected, the stationary phase data revealed a stronger response to 15 mM hydrogen peroxide exposure than what was observed previously by Cortes et al. (2020), who used 3 mM hydrogen peroxide. This is reflected by a doubled log_2_fold change range and nearly twice the number of DE genes. The most upregulated gene under these conditions was *glmS* with a log_2_fold change of 3.31. GlmS is an important enzyme for the synthesis of the peptidoglycan precursor UDP‐GlcNAc (L. Sun et al. 2021). In *Yersinia pestis*, UDP‐GlcNAc acts as a sugar donor for the *O*‐GlcNAcylation of proteins (Cao et al. 2024), a process used by both eukaryotes and prokaryotes in oxidative stress response (Cao et al. 2024; Groves et al. 2013). Additionally, upon deletion of *glmR*, which can act as a uridyltransferase enzyme to facilitate the production of UDP‐GlcNAc, increased levels of UDP‐GlcNAc were able to restore cell wall stress response in *L. monocytogenes* (Pensinger et al. 2023). These antioxidative functions and roles of UDP‐GlcNac suggest an indirect contribution of *glmS* to the *L. monocytogenes* oxidative stress response. *Bsh*, a virulence gene which was also upregulated under lactic acid stress, was the second most upregulated gene with a log_2_fold change of 3.08. The bile salt hydrolase encoded by *bsh* detoxifies bile salts found in the gastrointestinal tract of hosts. These bile salts induce oxidative damage to bacteria but can be deconjugated by the bile salt hydrolases (Begley et al. 2005). Although it has yet to be determined if *bsh* plays a role in the general oxidative stress response in *Listeria*, in a study using *Lacticaseibacillus casei* where *bsh* was coexpressed with a catalase gene, an improvement in general oxidative stress survival and bile salt resistance was observed (Wang et al. 2011). A σ^B^‐regulated conserved membrane protein encoded by *lmo0647* (*LM6179_*00957) was also one of the most upregulated genes with a log_2_fold change of 2.77.

Looking further at individual DE genes, many patterns arose that were similar to those observed in the lactic acid exposure gene expression results, albeit with some exceptions. Specific virulence genes were again upregulated in the stationary phase; in addition to *bsh*, *inlD* was upregulated with a log_2_fold change of 1.23. *Lmo2230* was also again significantly upregulated in the stationary phase with a log_2_fold change of 2.22. In addition, a peroxiredoxin (*ohrA*) and its transcriptional regulator (*ohrR*) were among the most upregulated genes with log_2_fold changes of 2.37 and 2.39, respectively. These results are consistent with the oxidative stress results observed by Cortes et al. (2020). Similarly, a previous study demonstrated that exposure of *L. monocytogenes* to chlorine dioxide results in the upregulation of *ohrA* (Pleitner et al. 2014). Additionally, OhrA was required for the regulation of virulence factors during infection, and disruption of *ohrA* affects the sensitivity of *L. monocytogenes* to peroxide and disulfide stress (Reniere et al. 2016). These findings demonstrate that OhrA and its regulator OhrR are important components of the oxidative stress response. Another similarity was observed in the upregulation of USP genes *lmo2673* and *lmo2748* (encoding GSP26) with log2fold changes of 1.51 and 1.63, respectively. Exposure to chlorine dioxide induced the upregulation of *lmo2673* (Pleitner et al. 2014), and deletion of the gene resulted in lessened survival to hydrogen peroxide, suggesting a role for *lmo2673* in *L. monocytogenes* oxidative stress response (Seifart Gomes et al. 2011). A direct relationship between GSP26 and oxidative stress response in *L. monocytogenes* has yet to be defined. However, when the ferritin‐encoding gene *fri* is deleted, *L. monocytogenes* cells become more sensitive to oxidative stress and GSP26 is upregulated in response, indicating an indirect involvement in oxidative stress response (Dussurget et al. 2005). GSEA results indicated the downregulation of pathways involved with chemotaxis, flagella biosynthesis, ribosomal proteins, and metabolism following both lactic acid and oxidative stress exposure (Figure A1 and Supporting Information Table A5).

When exposed to oxidative stress, *L. monocytogenes* engages in a survival strategy that typically involves the upregulation of catalase, superoxide dismutase, proteases, cold and heat shock proteins, and genes involved in damage repair (Manso et al. 2020; Wiktorczyk‐Kapischke et al. 2021). In this study, this response was observed but with relatively low gene expression changes. The catalase‐encoding gene *kat* (*lmo2785*; *LM6179_00206*) was significantly upregulated with a log_2_fold change of 1.00 in the stationary phase; however, superoxide dismutase and many cold and heat shock proteins had log_2_fold changes of less than 1. This may be attributed to the oxidative stress conditions chosen in this study, as a previous study using chlorine dioxide resulted in the upregulation of not only *kat*, but also superoxide dismutase and cold shock protein genes (Pleitner et al. 2014). Additionally, these differences may be affected by the concentration of hydrogen peroxide used. Cortes et al. (2020) used 3 mM hydrogen peroxide compared with the 15 mM used in this study and observed insignificant changes in the expression of *kat* and superoxide dismutase. It is possible that 15 mM hydrogen peroxide remains too low a concentration to elicit gene expression changes similar to those observed by Pleitner et al. (2014) in response to chlorine dioxide exposure. Notably, while the GAD system is most often associated with acid stress response, in stationary phase cells exposed to oxidative stress, the GAD system gene *gadD3* was one of the most upregulated genes with a log_2_fold change of 1.53. It has been previously demonstrated that the deletion of *gadD3* from *L. monocytogenes* results in decreased survival upon oxidative stress exposure (Boura et al. 2020). The same study indicated that *gadD3* plays a more prominent role in catalase activity, which may explain its upregulation following oxidative stress.

#### 3.2.2 Logarithmic Growth Phase Response to 15 mM Hydrogen Peroxide

The logarithmic growth phase cells had 819 significant DE genes with a range of −8.23 to 6.75 log_2_fold changes. Of these, 304 genes were upregulated and 515 were downregulated. The most upregulated gene in the logarithmic growth phase, *lmo0135*, encodes the cysteine transport protein CtaP and was upregulated with a log_2_fold change of 6.75. Other proteins involved in cysteine import, encoded by *lmo0136* and *lmo0137*, were also upregulated with log_2_fold changes of 6.03 and 5.30, respectively. Similarly, the *ytmI* operon (*lmo2343*–*lmo2352*), including its encoded cysteine/cystine transporter, TcyKLMN, was one of the most upregulated genes with a log_2_fold change range of 4.44–5.90. Notably, these genes were also upregulated in logarithmic cells exposed to 1% lactic acid. The upregulation of these transporters during oxidative stress indicates that, in addition to their role in acid stress, they are active in the oxidative stress response of logarithmic growth phase *L. monocytogenes* as well. This agrees with the assessment of Brenner et al. (2022) that transcription of the *ytmI* operon is affected by multiple environmental cues, including oxidative stress. This suggested role in oxidative stress could explain the attenuation of an *L. monocytogenes* mutant lacking *lmo0135*–*lmo0137* in Caco‐2 cells and mice (Schauer et al. 2010). Indeed, a role of cysteine in bacterial oxidative stress response has been discovered during the last few years (Tikhomirova et al. 2024). Cysteine acts as an antioxidant which scavenges free radicals in the cell (Guidea et al. 2020). Additionally, it is a precursor for the biosynthesis of glutathione, an important low molecular weight thiol used to repair oxidized proteins (Tikhomirova et al. 2024). The importance of cysteine to oxidative stress response has been demonstrated in a variety of bacteria, including *Neisseria meningitidis* (van de Waterbeemd et al. 2013), *Brucella ovis* (Varesio et al. 2021), and *Staphylococcus aureus* (Ji et al. 2012). Furthermore, in *Escherichia coli* the cysteine/cystine shuttle system as well as cysteine biosynthesis pathways have been highlighted as putative protective antioxidant mechanisms (Ohtsu et al. 2010; Roth et al. 2022). Therefore, the upregulation of cysteine importers may be an oxidative stress response in *L. monocytogenes*. Alternatively, the upregulation of these cysteine/cystine importers in the logarithmic growth phase relative to the stationary phase in both stress conditions may merely be reflective of the auxotrophy of *L. monocytogenes* for cysteine and its need to import it for growth (Berude et al. 2024).

**Figure 5:**
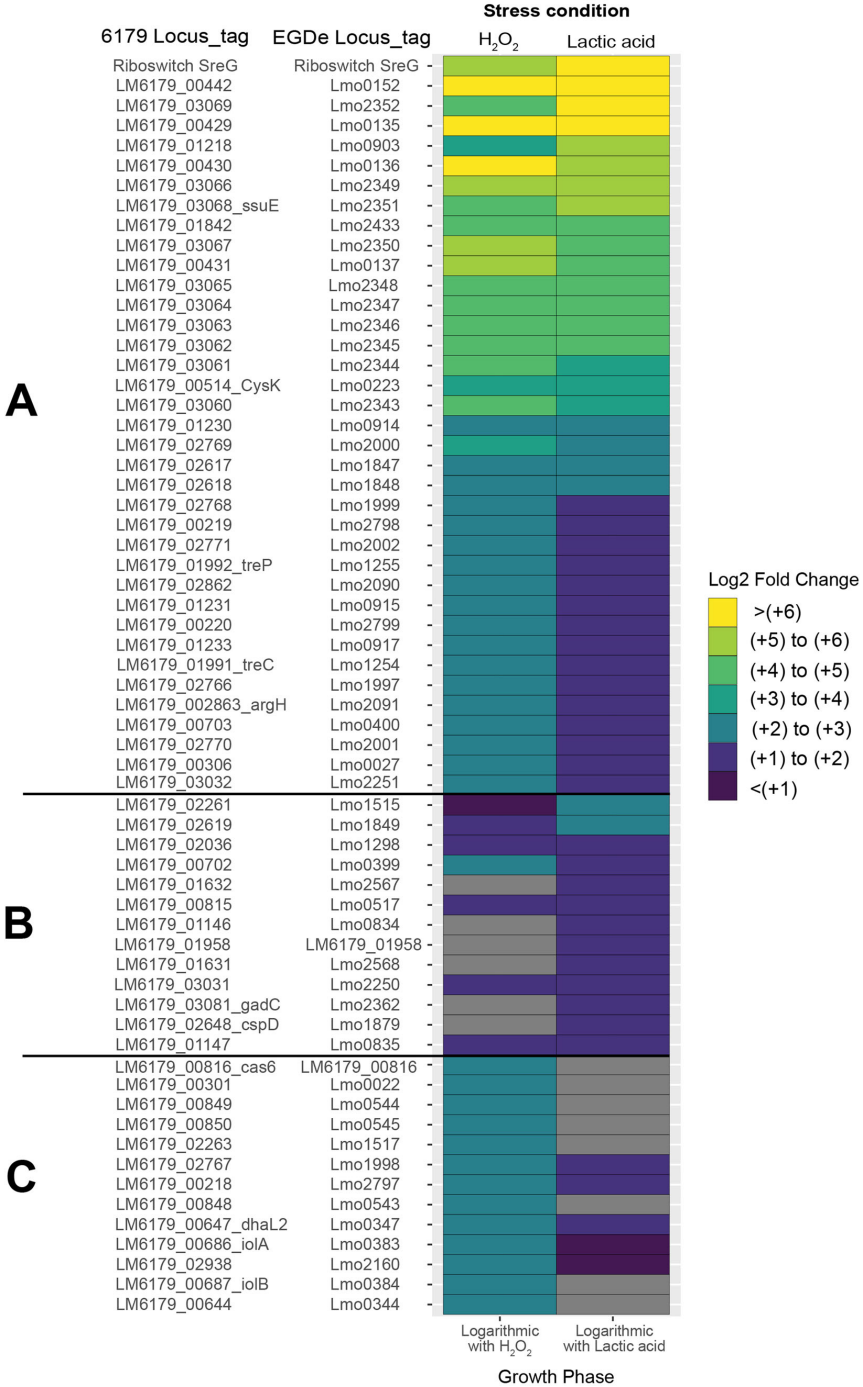
Heatmap of the 50 most upregulated significant (*Q* < 0.05 and log_2_fold changes with absolute values ≥ 1) DE chromosomal genes in logarithmic growth phase cells comparing their log_2_fold changes following exposure to 1% lactic acid and 15 mM hydrogen peroxide. Genes are designated with *Listeria monocytogenes* EGD‐e locus tags and *L. monocytogenes* 6179 locus tags, when applicable. Log_2_fold changes of these genes are displayed for logarithmic growth phase cells in response to both hydrogen peroxide and lactic acid. Genes shown in gray did not exhibit significant changes in expression. (A) Genes that belong to both the 50 most upregulated genes upon exposure to both hydrogen peroxide and to the 50 most upregulated genes upon exposure to lactic acid. (B) Genes which were only part of the set of 50 most upregulated genes upon lactic acid exposure. (C) Genes which were only part of the set of 50 most upregulated genes upon hydrogen peroxide exposure. DE, differentially expressed; EGD‐e, *L. monocytogenes* strain EGD‐e.

Analysis of the DE genes in logarithmic growth phase cells following oxidative stress highlighted an interesting pattern: 37 of the 50 most upregulated genes in response to hydrogen peroxide were also among the 50 most upregulated genes in response to lactic acid exposure (Figure 5 and Supporting Information Table A3). This observation suggests that the stress response of logarithmic growth phase cells may involve the upregulation of a shared set of genes. 17 of these 37 shared upregulated genes encode phosphotransferase systems (PTSs) involved in the transport of trehalose (*lmo1254* and *lmo1255*; *LM6179_01991* and *LM6179_01992*) (Ells and Truelstrup Hansen 2011; J. Wu et al. 2023b), mannitol (*lmo2797* and *lmo2799*; *LM6179_00218* and *LM6179_00220*), mannose (*lmo1997*–*lmo2002*; *LM6179_02766* – *LM6179_02771*), and lactose (*lmo0914–lmo0917*; *LM6179_01230*–*LM6179_01233*) (Stoll and Goebel 2010). In addition to log_2_fold changes, GSEA demonstrated enrichment of KEGG pathways related to PTS systems (Figure A1 and Supporting Information Table A5). The upregulation of PTS systems in stationary phase *L. monocytogenes* has been observed in response to acid stress (Horlbog et al. 2019), oxidative stress (Chen et al. 2023), and high‐pressure processing treatment (Duru et al. 2021), suggesting a role in general stress response. A regulatory relationship between glutathione and PTS systems in oxidative stress conditions has been recently identified, revealing that the overexpression of *lmo2002* of the mannose‐specific PTS system (*lmo1997–lmo2004)* leads to a decreased tolerance to oxidative stress conditions (Chen et al. 2023). However, in *Lactiplantibacillus plantarum*, impaired expression of mannose‐specific PTS systems resulted in increased peroxide sensitivity (Stevens et al. 2010). Together, these findings emphasize the need for controlled regulation of PTS systems for proper oxidative stress response. Regarding downregulated genes, tRNAs were the most downregulated with log_2_fold changes around –8.20. The remaining downregulated genes were comprised of CRISPR genes, ribonucleases, and proteins of unknown functions.

#### 3.2.3 Comparison of Logarithmic and Stationary Growth Phase Oxidative Stress Response Gene Expression Patterns

In comparison to lactic acid exposure, oxidative stress resulting from exposure to 15 mM hydrogen peroxide resulted in more DE genes and greater magnitudes of log_2_fold changes in logarithmic growth phase cells than in stationary phase cells (Table 2). Inversely expressed genes were once again observed, with 249 genes upregulated in one growth phase but downregulated in the other. This inverse expression and other contrasting results are made obvious when directly comparing the log_2_fold changes of the 50 most upregulated genes in the stationary phase in response to 15 mM hydrogen peroxide exposure to the log_2_fold changes of the same genes in the logarithmic growth phase (Figure 4A) and vice versa (Figure 4B). *Lmo2230* was inversely expressed, being downregulated in logarithmic phase with a log_2_fold change of −1.20 but upregulated in stationary phase with a log_2_fold change of 2.22. Further, only three of the 50 most upregulated genes in the logarithmic growth phase were regulated by σ^B^ compared with 31 genes in the stationary phase. Differences were also observed in the expression of GAD system genes. G*adD3* was upregulated with a log_2_fold change of 1.53 in the stationary phase but was not DE in the logarithmic growth phase. In contrast, *gadD2* was upregulated with a log_2_fold change of 1.45 in the logarithmic growth phase but had insignificant changes in the stationary phase. Both *gadD2* and *gadD3* have been shown to contribute to *L. monocytogenes* survival in oxidative stress conditions (Boura et al. 2020), although it remains unclear as to why the expression of the two genes differs between growth phases in this study.

In addition to the influence of the growth phase, the response of *L. monocytogenes* to oxidative stress is affected by other variables as well, making it difficult to draw broader conclusions across transcriptomics experiments with differing conditions. There are stark contrasts in stress response and gene expression between varying *L. monocytogenes* strains, as shown in previous studies (Cortes et al. 2020; Huang et al. 2018; Manso et al. 2020). For oxidative stress, the source of stress also impacts gene expression. A study using *L. monocytogenes* strain 6179 induced oxidative stress via cumene hydroperoxide (Harter et al. 2017). Their results showed significant increases in the expression of SSI‐2, but these genes were not DE in this study or the study by Cortes et al. (2020). A different study highlighted the importance of three USPs (*lmo0525*, *lmo1580*, and *lmo2673*; *LM6179_00830*, *LM6179_02331*, and *LM6179_00086*, respectively) in the *L. monocytogenes* EGD‐e oxidative stress response; however, both *lmo0525* and *lmo1580* were insignificantly changed or downregulated in this study (Seifart Gomes et al. 2011).

The differences in strain and peroxide treatment may also influence the results observed of genes encoding metalloregulatory proteins, such as *perR* (*lmo1683*; *LM6179_02439*) and *fur* (*lmo1956*; *LM6179_02726*). *PerR* has a role in peroxide response through the regulation of genes involved with iron homeostasis, such as *fur* (Rea et al. 2005; Ruhland and Reniere 2019); however, *perR* was insignificantly upregulated in stationary phase cells and downregulated with a log_2_fold change of −1.52 in logarithmic phase cells. *Fur*, an important iron homeostasis regulator needed to control iron intake during peroxide stress (Fuangthong et al. 2002), was upregulated in stationary phase cells with a log_2_fold change of 0.89 and downregulated in logarithmic growth phase with a log_2_fold change of −0.91. A similar occurrence is observed in the expression of redox homeostasis genes. Thioredoxin *trxA* (*lmo1233*; *LM6179_01541*), which is involved in oxidative stress regulation (J. Sun et al. 2019), as well as other genes encoding thioredoxin family proteins, were insignificantly DE in this study. The peroxidase‐encoding gene *ahpA* (*lmo1604*; *LM6179_02354*) has been found to be required for survival of acute peroxide stress (Cesinger et al. 2021), but is insignificantly expressed in this study, which may be due to the lower concentration of hydrogen peroxide and shorter exposure time used in our study. Additionally, the differential expression of the peroxiredoxin gene *prx* (*lmo1604*; *LM6179_02354*) was insignificant despite its implicated role in oxidative stress protection (Dons et al. 2014).

Temperature is also a critical component in oxidative stress response, as exposure of *L. monocytogenes* to oxidative stress at a lower temperature may result in not only different transcriptome patterns but higher survival compared with stress exposure at higher temperatures (Manso et al. 2020; Ochiai et al. 2017). Despite impaired survival at higher temperatures, genes involved in oxidative stress response, including *kat*, were induced more at 37°C than at 20°C in a study by Ochiai et al. (2017). Our study used a 20°C incubation temperature to better replicate the temperatures found in FPEs, which may explain the less dramatic gene expression changes observed for stationary phase cells.

### 3.3 Plasmid Gene Expression

In addition to chromosomal DE genes, we analyzed the expression of genes on the pLM6179 plasmid. It has been previously demonstrated that pLM6179 contributes to the tolerance to a multitude of stressors, including lactic acid, oxidative stress, and osmotic stress (Anast and Schmitz‐Esser 2021; Naditz et al. 2019). Similar to what was observed for chromosomal gene expression in response to oxidative stress, logarithmic growth phase cells had more DE genes with greater magnitudes of change in response to hydrogen peroxide; this was in contrast to the lactic acid data set, where stationary phase cells exhibited greater shifts in gene expression. Exposure to lactic acid resulted in 38 significant DE genes for stationary phase cells, comprised of 16 upregulated and 22 downregulated genes. Log_2_fold changes ranged from −5.45 to 2.73 (Table 3 and Supporting Information Table A4). In contrast, there were no significant plasmid DE genes in the lactic acid data set for logarithmic growth phase cells (Table 3 and Supporting Information Table A4). In response to hydrogen peroxide exposure, analysis identified DE genes in logarithmic growth phase cells. All nine of these genes were downregulated with log_2_fold changes ranging from −2.88 to −1.06 (Table 3 and Supporting Information Table A4). Stationary phase cells had six significant DE genes, two of which were upregulated and four of which were downregulated (Table 3 and Supporting Information Table A4). Despite the difference in the number of DE pLM6179 genes between the two growth phases, the directionality of the changes was more consistent than that observed in chromosomal genes, as none of the DE genes were inversely expressed between treatments.

**Table 3:** Number of significantly (*Q* < 0.05, log_2_fold changes with absolute values ≥ 1) differentially expressed (DE) pLM6179 genes following 1% lactic acid and 15 mM hydrogen peroxide exposure.

Growth phase, experimental condition	Number of DE plasmid genes	Number of upregulated DE plasmid genes	Number of downregulated DE plasmid genes	Log_2_fold change range
Stationary phase, 1% lactic acid	38	16	22	−5.45 to 2.73
Logarithmic growth phase, 1% lactic acid	0	0	0	N/A
Stationary phase, 15 mM hydrogen peroxide	6	2	4	−1.84 to 1.16
Logarithmic growth phase, 15 mM hydrogen peroxide	9	0	9	−2.88 to −1.06

#### 3.3.1 Plasmid Gene Expression Changes in Response to Lactic Acid Exposure

Intriguingly, in response to lactic acid exposure, only stationary phase cells upregulated pLM6179 genes. The most highly upregulated gene was *clpL* with a log_2_fold change of 2.72. In addition, *clpL* also had the highest expression level among all plasmid genes under all conditions. This is consistent with the findings of Cortes et al. (2020), who also identified *clpL* as the most upregulated pLM6179 gene in stationary phase cells in response to 1% lactic acid exposure. The *clpL* gene encodes a heat shock protein belonging to the AAA+ (ATPase associated with diverse cellular activities) protein family (Bohl et al. 2024) and has been shown to be involved in heat tolerance (Pöntinen et al. 2017). The upregulation of plasmid‐borne *clpL* has been recorded in response to lactic acid, low pH, and 6% salt (Cortes et al. 2020; Hingston et al. 2019). A large‐scale survey of plasmids in *L. monocytogenes* revealed the presence and broad distribution of *clpL* among plasmids of ST121 and many other STs from both lineage I and II *L. monocytogenes* (Schmitz‐Esser et al. 2021). These previous observations in combination with the significant upregulation of *clpL* in this study further suggest that ClpL may have a role in acid stress response. The remaining upregulated pLM6179 genes encode proteins with unknown functions and genes potentially involved in plasmid replication.

#### 3.3.2 Plasmid Gene Expression Changes in Response to Hydrogen Peroxide Exposure

Hydrogen peroxide exposure resulted in two upregulated pLM6179 genes in stationary phase cells with log_2_fold changes of 1.10 and 1.16. These genes encode a hypothetical protein and a protein involved in plasmid partitioning. Both of these genes were also upregulated by stationary phase cells in response to lactic acid stress. Logarithmic growth phase cells did not show any DE upregulated pLM6179 genes.

## 4 Conclusion

In this study, we observed distinct gene expression changes of *L. monocytogenes* 6179 during logarithmic and stationary growth phases in response to oxidative and lactic acid stress. We observed the conserved upregulation of 37 chromosomal genes in response to both stressors in logarithmic growth phase cells. These include genes involved in cystine transport, such as the *ytmI* operon (*lmo2343*–*lmo2352)* and genes *lmo0135*, *lmo0136*, and *lmo0137*. The conserved upregulation of cystine transport in response to both oxidative and acid stress suggests that this pathway is potentially involved more broadly in the stress response of logarithmic growth phase *L. monocytogenes*. Further, our results indicate that the stress response of stationary phase cells was more influenced by the type of stressor encountered. Compared with the logarithmic growth phase, the stationary phase gene expression changes more closely followed the canonical patterns associated with the acid response, such as the upregulation of the GAD and ADI system genes. Our results further emphasize the importance of σ^B^‐regulated genes in stress response and highlight the relationship between growth phase and the upregulation of σ^B^ genes. As discussed previously, multiple studies have noted the upregulation of *lmo2230* in response to stress exposure. Our results corroborate these findings by observing the upregulation of *lmo2230* in stationary phase cells in response to both acid stress and oxidative stress; however, the function and role of this gene in stress response have yet to be determined. These gene expression patterns, observed in both chromosomal and plasmid genes, contribute to the understanding of the impacts of growth phase on gene expression in *L. monocytogenes* in response to lactic acid and hydrogen peroxide stress exposure.

## Author Contributions

**Jessica L. Strathman‐Runyan:** data curation (lead), formal analysis (lead), visualization (lead), validation (lead), writing – original draft (equal), writing – review and editing (equal). **Bienvenido W. Tibbs‐Cortes:** conceptualization (equal), data curation, formal analysis, visualization, validation, writing – original draft, writing – review and editing. **Stephan Schmitz‐Esser:** conceptualization (equal), funding acquisition (lead), supervision (lead), visualization (supporting), writing – original draft (equal), writing – review and editing (equal).

## Ethics Statement

The authors have nothing to report.

## Conflicts of Interest

None declared.

## Supporting information


**Table A1:** DESeq2 results from cells in stationary phase exposed to either 1% lactic acid or 15 mM hydrogen peroxide.

**Table A2:** DESeq2 results from cells in logarithmic growth phase exposed to either 1% lactic acid or 15 mM hydrogen peroxide.

**Table A3:** The 37 genes upregulated in logarithmic growth phase cells in response to both 1% lactic acid and 15 mM hydrogen peroxide exposure.

**Table A4:** DESeq2 results for pLM6179 genes from cells in either stationary or logarithmic growth phase exposed to either 1% lactic acid or 15 mM hydrogen peroxide.

**Table A5:** Contains ClusterProfiler gene set enrichment analysis results for each of the four conditions analyzed in this study. For each significantly enriched KEGG pathway, the table lists its KEGG ID, its description, enrichment and normalized enrichment scores, statistical metrics, and leading edge analysis results.

## Data Availability

The data that support the findings of this study are openly available in NCBI SRA at https://www.ncbi.nlm.nih.gov/sra, reference number PRJNA1164155. The raw transcriptome sequencing data for this study were submitted to the National Center for Biotechnology Information (NCBI) Sequence Read Archive (SRA) under BioProject PRJNA1164155. The code used for this study can be found at https://github.com/benc347/rli47.
